# Infants of Mothers With Diabetes and Subsequent Attention Deficit Hyperactivity Disorder: A Retrospective Cohort Study

**DOI:** 10.3389/fped.2019.00452

**Published:** 2019-11-04

**Authors:** Chien-Heng Lin, Wei-De Lin, I-Ching Chou, Inn-Chi Lee, Syuan-Yu Hong

**Affiliations:** ^1^Division of Pediatrics Pulmonology, China Medical Univeristy Children's Hospital, Taichung City, Taiwan; ^2^Department of Biomedical Imaging and Radiological Science, College of Medicine, China Medical University, Taichung City, Taiwan; ^3^Department of Medical Research, China Medical University Hospital, Taichung City, Taiwan; ^4^Division of Pediatrics Neurology, China Medical Univeristy Children's Hospital, Taichung City, Taiwan; ^5^Graduate Institute of Integrated Medicine, College of Chinese Medicine, China Medical University, Taichung City, Taiwan; ^6^Department of Pediatrics, School of Medicine, Institute of Medicine, Chung Shan Medical University Hospital, Chung Shan Medical University, Taichung City, Taiwan

**Keywords:** infants, maternal diabetes, ADHD, attention deficit hyperactivity disorder, neurodevelopmental outcome

## Abstract

**Background:** Maternal diabetes mellitus (DM) increases the risk of fetal, neonatal, and long-term complications in offspring. Although this has been widely known for decades, data are limited regarding the effect of maternal pregestational and gestational diabetes on the subsequent neurodevelopmental outcome of offspring. This study investigated whether infants of mothers with diabetes (IDMs) were associated with a risk of subsequent attention deficit hyperactivity disorder (ADHD).

**Objectives:** We collected data from newborn infants born to mothers with gestational or pregestational diabetes at China Medical University Children's Hospital between January 1, 2006, and December 31, 2012. These patients were followed to evaluate their risk of ADHD (IDM group) compared with that for those born to mothers without DM (non-IDM group). Several assumed perinatal risk factors accompanying the IDMs were also analyzed.

**Results:** Overall, 104 patients with average gestational ages of 36.5 weeks were included in the IDM group. Additionally, 110 patients with average gestational ages of 36.6 weeks were included in the non-IDM group. Compared with non-IDMs (reference), the overall risk of ADHD in IDMs was 2.6 [95% confidence interval (CI)P, 1.11–5.90; *p* = 0.03]. Furthermore, the risk of ADHD among male (OR, 3.78; 95% CI, 1.37–10.3; *p* = 0.001) and full-term infants [odds ratio (OR), 4.5; 95% CI, 1.16–17.6; *p* = 0.03] in the IDMs was higher than that in the non-IDM group. No significant differences were found among IDMs for the assumed perinatal risk factors that were analyzed.

**Conclusions:** The study revealed a higher incidence rate of ADHD in IDMs, especially male and full-term infants. It is crucial for pediatricians to identify the early symptoms neurodevelopmental disorders, especially ADHD, in children of diabetic mothers to initiate proper assessment and treatment.

## Introduction

Maternal diabetes mellitus (DM) increases the risk of fetal, neonatal, and long-term complications in offspring. Although this has been widely known for years (1), publications regarding the effect of maternal pregestational and gestational diabetes mellitus (GDM) on the subsequent neurodevelopmental outcomes of offspring are still limited. Limited data suggest that infants of mothers with diabetes (IDMs) may have higher incidences of developing neurodevelopmental disorders (NDDs) (2, 3). However, the quality of evidence in these studies was insufficient because most of them did not adequately control for confounding factors associated with IDMs, such as maternal pre-pregnancy and neonatal morbidity. Likewise, evidence supporting the association between IDMs and attention deficit hyperactivity disorder (ADHD) remains inadequate (4, 5). Table 1 provides a summary of previous studies on NDDs in IDMs.

**Table 1:** Literature search and article analysis for neurodevelopmental disorders in infants of diabetic mothers.

**References**	**Research type**	**Case numbers**	**F/U years**	**Comments**
Ornoy et al. (6)	Case-control study	32	N/A	GDM induces long-term minor neurological deficits which are more pronounced in younger children
Ornoy et al. (7)	Case-control study	57	N/A	Pregestational or gestational diabetes was found to adversely affect attention span and motor functions of offspring at school age, but not their cognitive ability
Ornoy et al. (4)	Systematic review	N/A	N/A	Pregestational or gestational diabetes may adversely affect attention span and motor functions of the offspring, but not their cognitive ability unless complicated by nephropathy or hypertension
Mann et al. (8)	Retrospective cohort study	162, 611	3	Increased risk for ID among children born to mothers with DM
Cai et al. (5)	Retrospective study	74	2	GDM and maternal blood glucose levels are associated with offspring's attention problems later in life
Adane et al. (9)	Systematic review	N/A	N/A	Maternal DM during pregnancy was negatively associated with offspring's cognitive development
Wang et al. (10)	Meta-analysis	17 studies	N/A	GDM were associated with an increased risk of autism

*DM, diabetes mellitus; F/U, follow-up; GDM, Gestational diabetes mellitus; IDM, Infants of diabetic mothers; ID, Intellectual disability; N/A, Not applicable*.

ADHD is a disease with multifactorial etiology, characterized by symptoms of hyperactivity, impulsivity, or inattention that can affect cognitive, academic, behavioral, emotional, and social functioning (11). In addition to a heritable component, some early-life risk factors for ADHD that have been proposed include young maternal age, mothers with chemical intolerances, and prenatal exposure to smoking and alcohol (12, 13). This implies that the maternal and perinatal effects could occur early in life but become more serious later in life.

Based on the understanding of the neurodevelopmental influence of maternal DM and clinical observation of higher ADHD occurrence in IDMs, we conducted a retrospective cohort study to observe whether IDMs were associated with a risk of subsequent ADHD. Additionally, risk factors accompanying IDMs were investigated in this study. We hypothesized that IDMs have an increased risk of ADHD compared with infants of mothers without diabetes.

## Materials and Methods

### Patient Population

The study protocol was approved by the Ethics Review Board of China Medical University (Approval # CMUH108-REC1-023 and CMUH107-REC2-152). In this retrospective study, we collected hospitalization data on newborn infants born to mothers with gestational or pregestational diabetes at China Medical University Children's Hospital between January 1, 2006 and December 31, 2012. Assumed risk factors related to IDMs and for subsequent NDDs were as follows: prematurity, congenital anomalies or cardiomyopathy, perinatal insults (including fetal distress during labor, low 1-min Apgar score), macrosomia, and neonatal hypoglycemia. Preterm birth in the present study was defined as any birth before 37 completed weeks of gestation. Macrosomia was defined as birth weight over 4,000 g irrespective of gestational age. Neonatal hypoglycemia in the first 24 h of life was defined as a blood glucose level of <40 mg/dL. Congenital anomalies or cardiomyopathies were screened using echocardiography, cranial sonography, and renal ultrasound for each IDM by a neonatologist and confirmed by an individual subspecialist. The exclusion criteria were as follows:

1. Loss of contact with a patient during the follow-up period.
2. Patients who developed NDDs or epilepsy with documented etiology or followed by a causative event; for example, central nervous system infections, copy number variations, or single gene mutations, which were related to epilepsy and NDDs.
3. Patients who were born relatively preterm (<30 weeks).
4. Patients who had a history of traumatic brain injury during the follow-up period.
5. Maternal drug and alcohol use and smoking habit during pregnancy.

**Figure 1:**
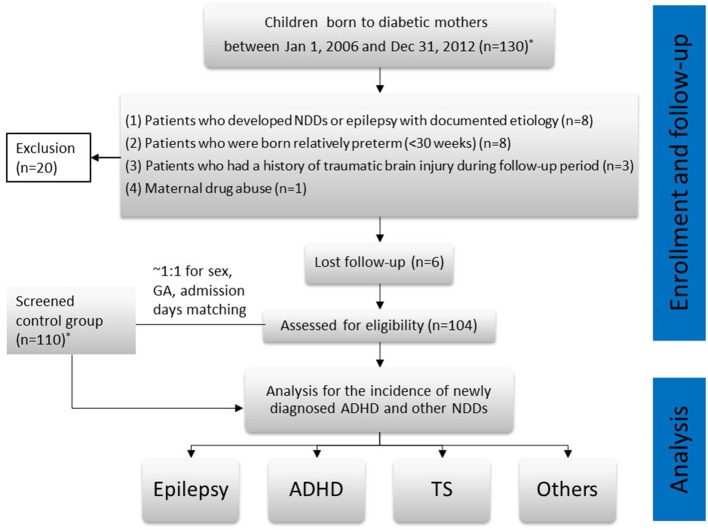
Flowchart of the study. ^*^The patients who were screened from the medical records and contacted by our case managers.

The final study population comprised 104 patients (IDM group). In addition, we selected 110 children who were immediately hospitalized to the sick baby room at birth for reasons other than those related to being IDMs (e.g., preterm birth; meconium aspiration syndrome, transient tachypnea of the newborn, suspected congenital infections) as controls. The exclusion and follow-up protocols for the control group and the IDM group were the same. The last patient was enrolled in December 2012. All patients included in the study were followed-up from baseline until December 31, 2018. Therefore, for the purposes of this study, the youngest patient diagnosed with ADHD was 6 years old. Quarterly, we followed-up patients by reviewing their medical records and contacting their families through telephone or e-mail from the beginning of 2016. ADHD in both groups was confirmed in two ways: (1) A definite diagnosis made by a pediatric psychiatrist or pediatric neurologist through a scientific assessment, which was documented in their medical records; and (2) Other children and their parents were contacted to return to our pediatric neurology clinic for completion of ADHD-specific scales that focused on the core symptoms of ADHD (14). Once ADHD was suspected, a comprehensive assessment was performed. Patients who met the relevant diagnostic criteria in the fourth and fifth editions of the Diagnostic and Statistical Manual of Mental Disorders (DSM-4, DSM-5) were diagnosed with ADHD. We compiled statistics and proceeded with the analysis to observe the incidence of ADHD and any other NDDs in the children. A flowchart for the study is illustrated in Figure 1.

### Statistical Analysis

All data were entered centrally. Categorical variables between groups were analyzed using χ2 tests. Furthermore, we calculated the incidence density rates of ADHD in both groups. The odds ratios (ORs) and 95% confidence intervals (CIs) were calculated to evaluate the associations between ADHD in the IDM group compared with the non-IDM group. In addition, univariate and multivariable analyses using logistic regression models were conducted to analyze the unadjusted and adjusted ORs and 95% CIs for the perinatal risk factors and maternal confounders for ADHD in the IDMs. All statistical analyses were performed using PASW Statistics version 18.0 (SPSS Inc., Chicago, IL, United States). In addition, for all statistical analyses executed, we considered a two-tailed *p*-value of <0.05 to be statistically significant.

## Results

### Data Analysis

Between January 1, 2006 and December 31, 2012, 214 children were enrolled in this study. Table 2 presents the participants' demographic factors. The participants' mean gestational age (GA) was 36.5 weeks (standard deviation = 2.12) in the IDM group and 36.6 weeks (standard deviation = 2.96) in the non-IDM group. The mean birth body weight (BBW) was 3,152 g (standard deviation = 782.6) in the IDM group and 2,599 gm (standard deviation = 709.5) in the non-IDM group. The proportion of boys was higher than that of girls in both groups. The assumed possible perinatal risk factors for the IDM group were the most common complications of prematurity in both groups; neonatal hypoglycemia (*n* = 13, 12.5%) and perinatal insults (*n* = 10, 9.1%) were the second most frequently noticed complications in the IDM and non-IDM group, respectively. The incidences of macrosomia and neonatal hypoglycemia in the IDM group were statistically different from those in the non-IDM group (*p* < 0.05). The incidences of associated NDDs were higher in IDMs than in non-IDMs in terms of ADHD and other childhood cognitive outcomes (Supplementary Table 1).

**Table 2:** Demographic and clinical characteristics of infants of diabetic mothers and a comparison with infants admitted to the sick baby room after birth.

	**Group**		
	***IDMs*, *n* = 104 (%)**	**Non-IDMs, *n* = 110 (%)**	***p***
**Gender (%)**			0.45
Male	61 (58.7)	70 (63.6)	
Female	43 (41.3)	40 (36.3)	
GA (mean) (SD)^*^	36.5 (2.12)	36.6 (2.96)	0.84
BBW (gm) (SD)^*^	3152 (782.6)	2599 (709.5)	<0.01
Days of admission (SD)^*^	8.44 (8.00)	8.57 (6.74)	0.9
Follow-up years (SD)^*^	8.32 (1.82)	8.27 (1.76)	0.82
**Reason for admission**	IDM	Non IDM^†^	
**Possible perinatal risk factors (%)**			
Prematurity	39 (37.5)	40 (36.3)	0.9
Congenital anomalies (including cardiomyopathy)	5 (4.8)	4 (3.6)	0.66
Perinatal insults (including fetal distress during labor, low 1-min Apgar score)	7 (6.7)	10 (9.1)	0.68
Macrosomia	9 (8.6)	6 (5.4)	0.03
Neonatal hypoglycemia	13 (12.5)	4 (3.6)	0.02
**Neurodevelopmental disabilities (%)**			
ADHD	20 (19.2)	9 (8.2)	0.02
Tourette's syndrome	4 (3.8)	2 (1.8)	0.25
Epilepsy	2 (1.9)	4 (3.6)	0.12
Other negative childhood cognitive outcomes^‡^	24 (23.0)	12 (10.9)	0.03

* *t-test; BBW, birth body weight; GA, gestational age*.

† *Non-IDM: prematurity (n = 40), meconium aspiration syndrome (n = 12), congenital infection (n = 12), transient tachypnea of the newborn and low grade respiratory distress syndrome (n = 20) and others (n = 26)*.

‡ *Other negative childhood cognitive outcomes documented in patient medical records included intellectual disability; autism spectrum disorders; developmental coordination disorder and stereotypic movement disorder; developmental language disorder; and behavioral disorders including conduct disorder*.

### Difference in ADHD Incidence Between the IDMs and Non-IDMs

Table 3 compares the incidence rates and relative risks of ADHD for the IDMs and non-IDMs. The overall ADHD risk in the IDM group was higher than that in the non-IDM group (adjusted OR, 2.56; 95% CI, 1.11–5.90; *p* = 0.03). Furthermore, the risk of ADHD among male patients in the IDM group was even higher than that in the non-IDM group (Adj. OR, 3.78; 95% CI, 1.37–10.3; *p* = 0.001). In addition, full-term IDMs had a 4.5 times (95% CI, 1.16–17.6; *p* = 0.03) higher risk of having ADHD compared with those in the non-IDM group.

**Table 3:** Incidence rates and relative risks of attention deficit hyperactivity disorder for the infants of diabetic mothers group and infants without diabetic mothers groups and those stratified by sex and term and preterm neonates by using a logistic regression model.

**Group**	**ADHD**			
	**Event (No.)**	**IR (%)**	**OR (95% CI)**	**Adj. OR (95% CI)**
**Non-IDMs (*****n*** **=** **110)**	9	8.1	Reference^†^	Reference^†^
**Sex**				
M (*n* = 70)	7	10	Reference^†^	Reference^†^
F (*n* = 40)	2	5	Reference^†^	Reference^†^
**Gestation**				
Full term (*n* = 70)	3	4.2	Reference^†^	Reference^†^
Preterm (*n* = 40)	6	15	Reference^†^	Reference^†^
**IDMs (*****n*** **=** **104)**	20	19.2	2.30 (1.03, 5.16)^*^	2.56 (1.11, 5.90)^*^
**Sex**				
M (*n* = 61)	16	26.2	3.2 (1.21, 8.41)^**^	3.78 (1.37, 10.3)^**^
F (*n* = 43)	4	9.3	1.95 (0.34, 11.2)	1.94 (0.32, 11.7)
**Gestation**				
Full term (*n* = 65)	10	15.3	4.06 (1.06, 15.5)^*^	4.52 (1.16, 17.6)^*^
Preterm (*n* = 39)	10	25.6	1.95 (0.63, 6.03)	2.02 (0.651, 6.30)

*Adj OR, adjusted odds ratios IR, Incidence rate; CI, Confidence interval; M, male; F, female. Model adjusted by gestational age, birth body weight, days of hospitalization*.

† *Reference in each subgroup of the non-IDMs denotes a baseline for comparison of that in the IDMs*.

* *p <0.05*.

** *p <0.01*.

Table 4 compares the assumed risk factors in IDMs with the development of ADHD. No significant differences were observed among IDMs despite the number of risk factors they had or different maternal confounders (method of DM control; type of DM). Distribution of assumed perinatal risk factors (number 1–4) of IDMs and the respective number of patients are shown in Supplementary Table 2.

**Table 4:** Relative risks of attention deficit hyperactivity disorder for infants of diabetic mothers with different numbers of perinatal risk factors and maternal confounders and their baseline.

**Group**	**ADHD**	
**IDMs (*****n*** **=** **104)**	**OR (95% CI)**	***p***
**Number of assumed perinatal risk factors**^†^^‡^		
0	Reference	
1	0.78 (0.11, 5.04)	0.78
2	3.75 (0.73, 19.3)	0.11
3	5.40 (0.69, 41.7)	0.10
4	9.00(0.78, 103.7)	0.78
**Maternal confounders**		
DM controlled with diet	Reference	
DM controlled with insulin	0.86 (0.32, 2.28)	0.76
Gestational DM	Reference	
Pregestational DM	1.00 (0.32, 3.08)	0.99

*OR, odds ratio, DM, diabetes mellitus*.

† *Assumed perinatal risk factors: Prematurity, congenital anomalies, or cardiomyopathy, perinatal insults (including fetal distress during labor, low 1-min Apgar score), macrosomia, neonatal hypoglycemia*.

‡ *Breaking down of assumed perinatal risk factors (Number 1–4) of IDMs and the respective number of patients are shown in Supplementary Table 2*.

## Discussion

Although it is not a new concern that IDMs are subject to certain NDDs during childhood, relevant studies are limited (3, 15). The major challenges for studies have been short follow-up periods and the non-specific investigation of a single NDD. Childhood NDDs have a high age correlation and require specific assessment tools for examining each disorder. Because children are not subject to a diagnosis of ADHD until the age of 6 years, a short follow-up period for IDMs is not sufficient to obtain a complete understanding of their neurodevelopmental outcomes. Therefore, we investigated the incidence of NDDs and focused on ADHD in IDMs over a longer period until children were aged 6–10 years. In addition, we examined whether possible risk factors exist that may trigger the development of ADHD in these children. In our study, we showed that maternal GDM and pregestational DM had an identical incidence rate of ADHD in offspring, which was 2.6 times that of the non-IDM group. Whether maternal GDM or pregestational DM lead to DM in offspring that further contributes to ADHD in adolescents or young adults falls beyond the scope of this study because of a too short follow-up period, but is, however, worthy of careful consideration in future research. From a review of the literature, we observed that maternal GDM is negatively associated with offspring's childhood cognitive development, particularly language development (16). Additionally, other NDDs, such as autism and intellectual disability (ID), were mentioned in relation to maternal DM in some studies (6, 8–10). Although rare, ADHD has also been reported in relation to maternal DM in some studies (4, 5, 7).

Common neonatal morbidity factors associated with IDMs that we also assumed to be risk factors for ADHD included prematurity, congenital anomalies, perinatal insults (including fetal distress during labor, low 1-min Apgar score, and intrauterine death), macrosomia, neonatal hypoglycemia, and cardiomyopathy (17). However, when we investigated these factors among the IDM group, surprisingly, no significant differences were found in the occurrence of ADHD. Consequently, we proposed two plausible explanations. First, prognostic factors related to neurodevelopmental outcomes in IDMs were probably more complex and interactive; therefore, the exact effect of diabetic fetopathy on neural development was unclear. Although some studies have proposed that iron deficiency resulting from the rapidly expanding red cell mass in developing organs may contribute to adverse brain development (18–20), a single risk factor should not be taken as an independent factor. Ornoy et al. investigated various NDDs and the severity of perinatal complications associated with IDMs and did not detect a direct relation between the two (6). Second, in addition to perinatal factors, an adverse maternal physiologic environment caused by diabetes, including oxidative stress, inflammation, and maternal hyperglycemia, can also be associated with abnormal fetal brain development. Therefore, more studies starting early in pregnancy should be performed to examine the links between prenatal stress, perinatal complications, offspring behavioral and psychological problems, and susceptibility to ADHD (21, 22). Although the higher prevalence of ADHD in boys than in girls is unsurprising (23), our results revealed that this trend could be more conspicuous in IDMs. However, although many studies have suspected that ADHD is correlated to preterm, especially early preterm (24, 25), one unexpected result was that in IDMs, full-term infants appeared to have a higher risk of ADHD than premature infants. Likewise, a study investigated extremely preterm infants (GA 22–28 weeks) born between 2006 and 2011 and detected no difference in neurodevelopmental outcomes among the initial three study groups (infants born to mothers who used insulin before pregnancy, infants born to mothers who started using insulin during pregnancy, and infants born to maternal controls) at 18–22 months corrected age (9). We are unsure whether a longer gestational period indicates a more serious effect of diabetic fetopathy or what actual effect diabetes during pregnancy has on preterm infants; additional research is required to explore this phenomenon. We suggest that ADHD might be a long-term neurodevelopmental complication for IDMs. Among the participants, male and full-term infants had higher risks of developing ADHD; thus, these characteristics should be considered as potential risk factors within such groups. However, a more rigorous study that focuses on ADHD and motor and cognitive function in IDMs is essential in the future.

Studies have showed that IDMs have an increased lifetime risk of diabetes. The risk of DM (either T1DM or T2DM) in an offspring of a DM mother arises from both genetic susceptibility (26–30) and intrauterine environmental factors (31). A rigorous study reported that DM was detected in 21% of offspring of women with GDM and 11% of offspring of women with T1DM, compared with 4% of offspring of women from the background population. These findings implied a combined effect of maternal hyperglycemia and other factors on the risk of offspring developing diabetes. However, more specific risk factors underlying pathogenic mechanisms remain unknown (31).

This study had some limitations. First, although we detected a possible risk of ADHD in IDMs through this observational study, more in-depth investigations are expected in the future to determine associated risks and to identify their underlying mechanisms. As stated above, some confounding factors may have affected the results, such as the health and diabetes control status of the mother before and during pregnancy, socioeconomic status, pharmacological treatment of children before preschool age, malnutrition, and other environmental factors. Second, our sample size was not sufficiently large; thus, a population based cohort study should be designed to prove our findings. Third, we did not perform an analysis of ADHD subtype because of study design restrictions. Fourth, the sample was biased because it had a higher number of boys than girls, given that boys may actually have a higher occurrence of ADHD in the general population, and this may have influenced the outcome of risk calculations. Furthermore, there are some difficulties for our researchers to convince large enough families of healthy babies to return to the hospital for completion of ADHD-specific scales. On the contrary, babies who discharged from the sick baby room underwent a routine follow-up till school age based on hospital policy. Therefore, additional research that focuses on neuroimaging, genetic susceptibility, iron homeostasis, or proinflammatory cytokines, and a control group based on healthy babies may identify potential mechanisms.

In summary, the results of this study revealed a higher incidence rate of ADHD in children who were IDMs, especially male and full-term infants. It is crucial for pediatricians to identify the early symptoms of NDDs, especially ADHD, in children who were IDMs to initiate proper assessment and treatment. Additional patient data and more detailed studies are warranted to elicit potential prenatal, perinatal, postnatal, and maternal confounders in order to clarify the influence of being born to a mother with diabetes and its association with ADHD.

## Data Availability Statement

The datasets generated for this study are available on request to the corresponding author.

## Ethics Statement

After a full description of the study, written informed consent of participation was obtained from the legal guardians. The study protocol was approved by the Ethics Review Board of the China Medical University ethics committee (Approval #CMUH108-REC1-023 and # CMUH107-REC2–017 and #CRS-106-027 and #CRS-106-031).

## Author Contributions

S-YH collected and analyzed the data and prepared the draft. C-HL and W-DL participated in the design of the study and wrote the manuscript. I-CL and I-CC compiled the statistics of this study and participated in the editing and revising the tables. All authors read and approved the final manuscript.

### Conflict of Interest

The authors declare that the research was conducted in the absence of any commercial or financial relationships that could be construed as a potential conflict of interest.
