# Supplementary material for: Infants of Mothers With Diabetes and Subsequent Attention Deficit Hyperactivity Disorder: A Retrospective Cohort Study
Source: Front Pediatr. 2019 Nov 4;7:452. doi: 10.3389/fped.2019.00452 (PMC6844289; doi:10.3389/fped.2019.00452)
Supplement: Supplementary file 1 [file Data_Sheet_1.PDF]

Supplementary Table 1: All neurodevelopmental disorders (NDDs) detected in the IDM group.

	NDDs											
		Epilepsy	TS	ASD	ID	DCD	Learning problems	DLD	Behavioral disorders	Epilepsy +ASD	DLD+TS	DLD+ID
Only ADHD	9	-	-	-	-	-	-	-	-	-	-	-
ADHD plus		-	1	2	2	1	2	2	-	1	-	-
Only 1 non-ADHD NDD		1	2	2	4	-	3	7	1	-	-	-
≧2 non-ADHD NDDs		-	-	-	-	-	-	-	-	-	1	3

[*]**The numbers in the table refer to the number of patients, “-“ refers to “no patient”**

[†]ADHD, attention deficit hyperactivity disorder; autism spectrum disorders; DCD, Developmental coordination disorder; DLD, Developmental Language Disorder; ID, Intellectual disability; TS, Tourette syndrome

Supplementary Table 2: Distribution of assumed perinatal risk factors (number 1–4) of IDMs and the respective number of patients.

Assumed perinatal risk factors and their respective No. of patients		A	B	C	D	E
**1 assumed risk factor**						
**No.**	**12**	+				
	**5**					+
	**3**			+		
	**14**					+
	**5**		+			
**2 assumed risk factors**						
**No.**	**4**	+			+	
	**3**	+		+		
	**1**			+	+	
	**9**	+				+
	**4**				+	+
	**4**	+	+			
	**1**		+		+	
	**2**			+		+
	**4**		+	+		
	**2**		+			+
**3 assumed risk factors**						
**No.**	**1**	+		+		+
	**2**			+	+	+
	**1**	+	+	+		
	**1**		+		+	+
	**1**	+		+	+	
	**1**	+			+	+
	**1**	+	+		+	
**4 assumed risk factors**						
**No.**	**1**	+		+	+	+
	**2**		+	+	+	+
	**1**	+	+		+	+

A: Prematurity, B: congenital anomalies or cardiomyopathy, C: perinatal insults (including fetal distress during labor, low 1-minute Apgar score), D: macrosomia, E: neonatal hypoglycemia
